# Whole-genome-based phylogeny of *Bacillus cytotoxicus* reveals different clades within the species and provides clues on ecology and evolution

**DOI:** 10.1038/s41598-018-36254-x

**Published:** 2019-02-13

**Authors:** Marc J. A. Stevens, Taurai Tasara, Jochen Klumpp, Roger Stephan, Monika Ehling-Schulz, Sophia Johler

**Affiliations:** 1 Institute for Food Safety and Hygiene, Vetsuisse Faculty, University of Zurich, Zurich, Switzerland; 2 Institute for Food, Nutrition and Health, ETH Zurich, Zurich, Switzerland; 3 Functional Microbiology, Institute of Microbiology, Department of Pathobiology, University of Veterinary Medicine Vienna, Vienna, Austria

## Abstract

*Bacillus cytotoxicus* is a member of the *Bacillus cereus* group linked to fatal cases of diarrheal disease. Information on *B. cytotoxicus* is very limited; in particular comprehensive genomic data is lacking. Thus, we applied a genomic approach to characterize *B. cytotoxicus* and decipher its population structure. To this end, complete genomes of ten *B. cytotoxicus* were sequenced and compared to the four publicly available full *B. cytotoxicus* genomes and genomes of other *B. cereus* group members. Average nucleotide identity, core genome, and pan genome clustering resulted in clear distinction of *B. cytotoxicus* strains from other strains of the *B. cereus* group. Genomic content analyses showed that a hydroxyphenylalanine operon is present in *B. cytotoxicus*, but absent in all other members of the *B. cereus* group. It enables degradation of aromatic compounds to succinate and pyruvate and was likely acquired from another *Bacillus* species. It allows for utilization of tyrosine and might have given a *B. cytotoxicus* ancestor an evolutionary advantage resulting in species differentiation. Plasmid content showed that *B. cytotoxicus* is flexible in exchanging genes, allowing for quick adaptation to the environment. Genome-based phylogenetic analyses divided the *B. cytotoxicus* strains into four clades that also differed in virulence gene content.

## Introduction

*Bacillus cytotoxicus* was first described in 2013 as a thermotolerant member of the *Bacillus cereus* group^1^. At that time, only five strains had been detected. Four out of the five strains had been linked to severe foodborne diarrheal outbreaks, which included three fatal cases^1^. The *B. cereus* group has been rapidly expanding in recent years^1–6^ and comprises several genetically closely related species. Its most prominent members are *Bacillus anthracis*^7^, *Bacillus cereus sensu stricto*^8^, *B. cytotoxicus*^1^, *Bacillus mycoides*^9^, *Bacillus pseudomycoides*^10^, *Bacillus thuringiensis*^11^, *Bacillus toyonensis*^12^, and *Bacillus weihenstephanensis*^13^. A clear phylogenetic separation of *B. cereus* group species is not possible as the criteria that define species were not phylogenetically based^14–16^. However, three major clades can be differentiated within the *B. cereus* group, in which species are intermingled^17^. The evolution of the *B. cereus* group was recently suggested to be mainly driven by the adaptation to animal hosts as pathogens, symbionts, or saprophytes^18^. The enormous variation in pathogenic potential shown by members of this group is often linked to plasmid-encoded virulence factors. While in particular *B. anthracis* and *B. cereus* are frequent causes of morbidity and mortality, other *B. cereus* group species are used as probiotics or biopesticides. Still, the virulence potential of *B. cytotoxicus, B. thuringiensis* and *B. weihenstephanensis* may need to be reassessed based on new genomic data as well as data confirming the formation of various toxins^19–23^.

*B. cytotoxicus* was first isolated in association with three fatal cases of diarrheal disease in a foodborne outbreak in France in 1998^24^. The implicated causative agent, strain NVH 391–98^T^, produced cytotoxin K, a novel diarrheic enterotoxin, and was first identified as *B. cereus*^24^. However, several years later, multi-locus sequence typing and 16S rRNA sequence comparisons showed that NVH 391-98^T^ belonged to the novel species *B. cytotoxicus*^1^. This species characteristically harbors the *cytK-1* variant of the gene encoding cytotoxin K, which is highly toxic to human intestinal Caco-2 and Vero cells^25^. Homologues of CytK-1 with up to 89% amino acid identity were found in other *B. cereus* group members and are referred to as CytK-2^25^. *B. cytotoxicus* was described as a novel species in 2013 based on five strains, four of which were linked to foodborne disease^1^. However, it has been known for some time that cytotoxicity of *B. cytotoxicus* strains varies, with strain NVH 883-00 having been reported to be non-cytotoxic^26^. We were recently able to show that cytotoxicity of nine *B. cytotoxicus* isolates obtained from mashed potato powders varied greatly in a Vero cell assay^23^. While either no (n = 7) or low cytotoxicity (n = 1) was detected for most isolates, one isolate exceeded the cytotoxitiy of reference strain *B. cereus* NVH 0075-95, which was linked to foodborne disease^23^, by more than 3-fold.

The *B. cereus* group can be divided into three clades. Assignment of strains to clades can be performed using *spoIIIAB* typing^27,28^. *B. cereus* group strains can be further differentiated into seven phylogenetic subtypes^29^, to which new strains can be assigned by *panC* typing^30^. *B. cytotoxicus* strains are exclusively assigned to *panC* group VII. *B cytotoxicus* cannot only be differentiated from other *B. cereus* group members by its *panC* sequence, but also by the presence of the *cytK*-1 gene and its capability to grow at 50 °C^1,31^. Since the isolation of French outbreak strain NVH 391-98^T^, a small number of other *B. cytotoxicus* have been isolated, mainly originating from potato products and in particular mashed potatoes^1,23,32^.

Nevertheless, little is known about the population structure, ecology, and evolution of *B. cytotoxicus* and only four complete genome sequences were publicly available. In this study, we aimed to extend the genomic information available for this species. We generated complete genome sequences for ten *B. cytotoxicus* strains and studied the phylogeny and diversity of *B. cytotoxicus* strains. In addition, *B. cytotoxicus-*specific genetic traits were determined by comparison with genomes of other *B. cereus* group members, and plasmids present in the *B. cytotoxicus* strains were analyzed. The generated data significantly extends the very limited body of knowledge on *B. cytotoxicus*, in particular allowing for novel insights into the evolution and differentiation of this species.

## Results

### Species confirmation and position of *B. cytotoxicus* within the *B. cereus* group

*B. cytotoxicus* is phylogenetically different from other *B. cereus* group species based on 16S rRNA gene sequence similarity, MLST profile, and *panC* sequence^1,31^. The four publicly available genomes as well as the ten newly generated complete genomes (Table 1) were checked for correct species annotation using BTyper^31^. All 14 strains were placed into *panC* group VII of the *B. cereus* group^17^ based on *panC* sequence homology (Table 1). This confirms the identification of the strains as *B. cytotoxicus*.

**Table 1:** List of strains.

Species	Strain	Accession number	*panC* group
*B. cytotoxicus*	CH_1	CP024120	VII
	CH_2	CP024116	VII
	CH_3	CP024113	VII
	CH_4	CP024111	VII
	CH_13	CP024109	VII
	CH_15	CP024107	VII
	CH_23	CP024104	VII
	CH_25	CP024101	VII
	CH_38	CP024098	VII
	CH_39	CP024096	VII
	NVH 391-98^T^	NC_009674	VII
	NVH 883-00	NZ_FMJN00000000	VII
	CVUAS_2833	NZ_JYPG01000012	VII
	AFSSA_08CEB44bac	NZ_FMIK00000000	VII
*B. anthracis*	Ames_Ancestor	NC_007530	III
	Ames	NC_003997	III
	CDC684	NC_012581	III
	H9401	CP002091	III
	Sterne	AE017225	III
*B. cereus*	AH187	CP001177	III
	ATCC10987	NC_003909	III
	ATCC14579^T^	NC_004722	IV
	B4264	CP001176	IV
	E33L	NC_006274	III
	G9842	CP001186	IV
	Q1	NC_011969	III
*B. mycoides*	ATCC 6462	NZ_CP009692	VI
*B. pseudomycoides*	DSM 12442^T^	ACMX01000000	I
	FSL H8-0534	MUAQ01000000	I
*B. thuringiensis*	BMB171	NC_014171	IV
	YBT 1518	NC_022873	IV
	ser_konkukian_97_27	NC_005957	III
*B. weihenstephanensis*	KBAB4	NC_010184	VI
	WSBC10204^T^	NZ_CP009746	VI
*B. toyonensis*	BAC3151 BCT 7112^T^	LDKD02000000	V
		CP006863	V

**Figure 1:**
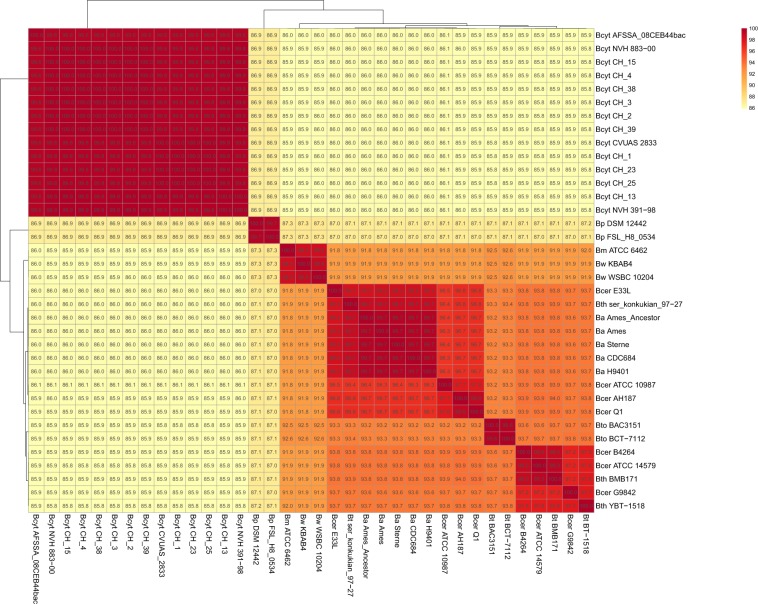
Whole-genome-based analysis of *B. cytotoxicus* compared to *B. cereus sensu lato*. Heat map of the average nucleotide identity (ANI) among the 37 strains. The percentages are listed in the figure and are also available as Supplementary Table S1. BA = *Bacillus anthracis*, Bcyt = *B. cytotoxicus*, BM = *B. mycoides*, Bp = *B. pseudomycoides*, Bcer = *B. cereus*, BW = *B. weihenstephanensis*, Bth = *B. thuringiensis*, Bto = *B. toyonensis*.

**Figure 2:**
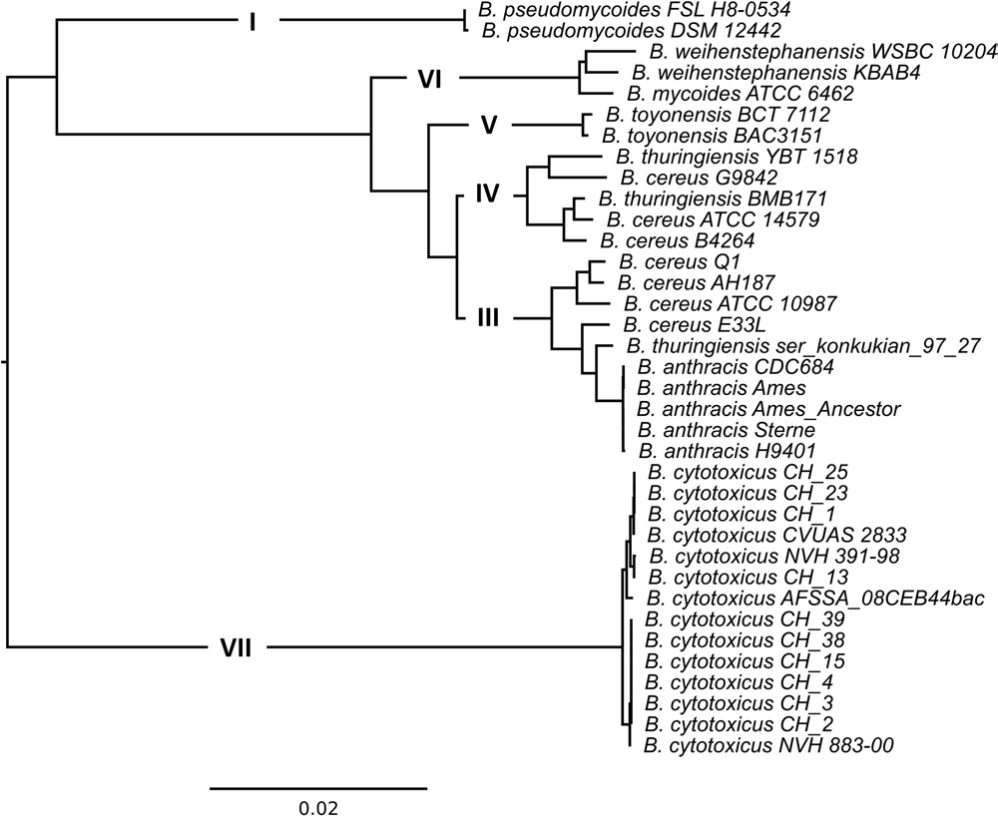
Neighbor-joining mid-point-rooted tree of the concatenated core proteins of the 37 strains. The clades as predicted by *panC* analysis are displayed in the tree.

**Figure 3:**
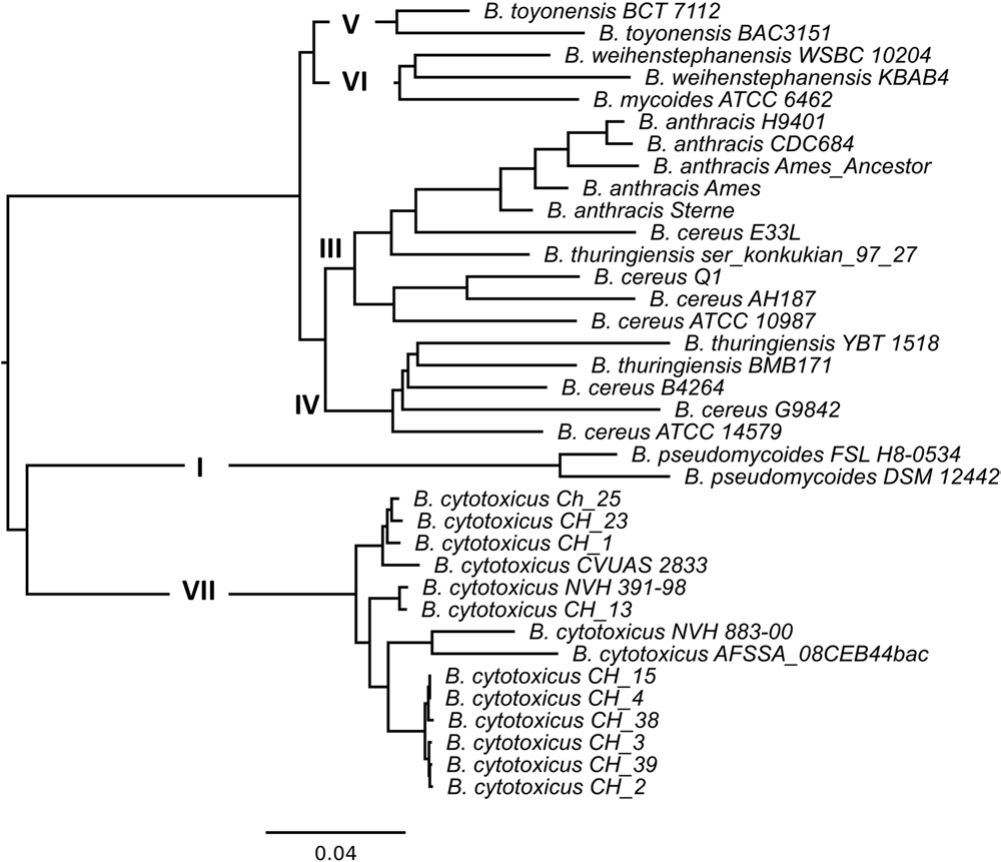
Hierarchical clustered mid-point-rooted tree based on the pan genome of 37 strains from the *B. cereus* group. The clades as predicted by *panC* analysis are displayed in the tree.

To check the whole-genome-sequence-based position of *B. cytotoxicus* within the *B. cereus* group, we compared the 14 genomes with selected strains from the *B. cereus* group. The average nucleotide identity (ANI) between all pairs of *B. cytotoxicus* strains was at least 99.3%, showing that *B. cytotoxicus* is a homogeneous species (Fig. 1). *B. cytotoxicus* has an approximate 85% ANI when compared to other *B. cereus* group members, with the highest value of 86.9% with *B. pseudomycoides*. Similar to *B. cytotoxicus*, *B. pseudomycoides* had an ANI of approximately 86% when compared to other *B. cereus* group members. As the ANI among the other members of the *B. cereus* group was at least 90% (Fig. 1), *B. cytotoxicus* and *B. pseudomycoides* are the most distinct members of the *B. cereus* group. The 14 *B. cytotoxicus* strains also form distinct clades in trees based on the core and pan genome of the 37 strains (Figs 2 and 3).

### Phylogenetic analysis of the species *B. cytotoxicus*

While the species *B. cytotoxicus* seems homogenous in the core genome analysis, the individual strains appeared to be more divergent from one another in the pan genome analysis. In particular, a shift in position was observed for strain NVH 883-00 (Figs 2 and 3). Therefore, we evaluated the phylogeny of the 14 *B. cytotoxicus* genomes more closely.

**Figure 4:**
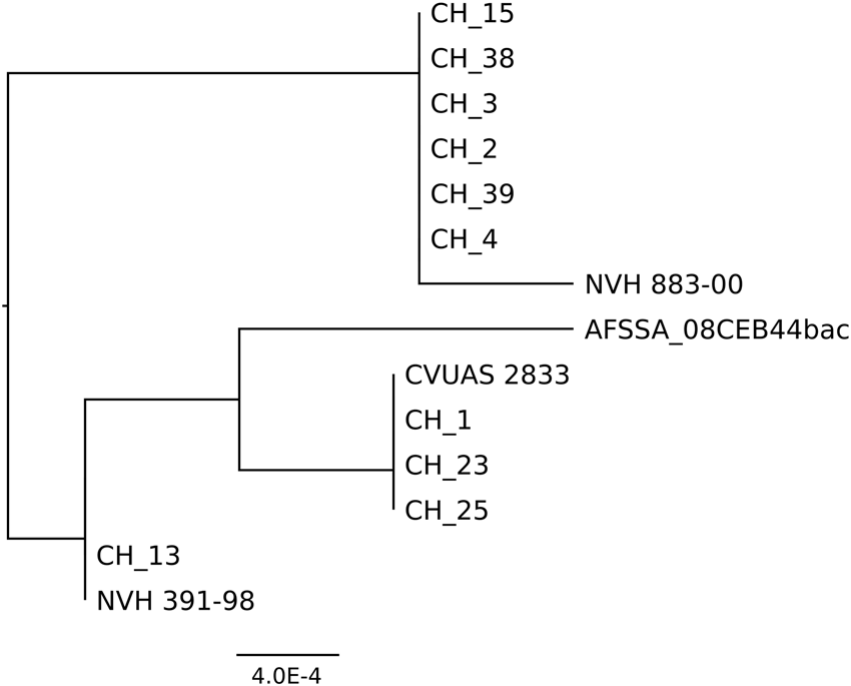
Neighbor-joining tree for strains of the species *B. cytotoxicus*. The tree is based on the alignment of the concatenated sequences of the MLST genes *glp*, *gmk*, *ilv*, *pta*, *pur*, *pyc*, and *tpi*.

First, we selected the sequences of the seven MLST genes commonly used for *B. cereus* typing (*glp*, *gmk*, *ilv*, *pta*, *pur*, *pyc*, and *tpi*)^33^. The sequences of *glp*, *gmk*, and *pyc* were 100% identical among the 14 *B. cytotoxicus* strains. Two different alleles occurred for *ilv*, *pur* and *tpi*, and three different alleles for *pta*. A tree based on the concatenated sequences of the seven genes revealed a close relation between strains CH_1, CH_23, CH_25 and CVUAS 2833, between strain CH_13 and the type strain NVH 391-98, and between strains NVH 883-00, CH_2, CH_3, CH_4, CH_15, CH_38 and CH_39 (Fig. 4). Strain AFSSA_08CEB44bac formed a clade of its own.

**Figure 5:**
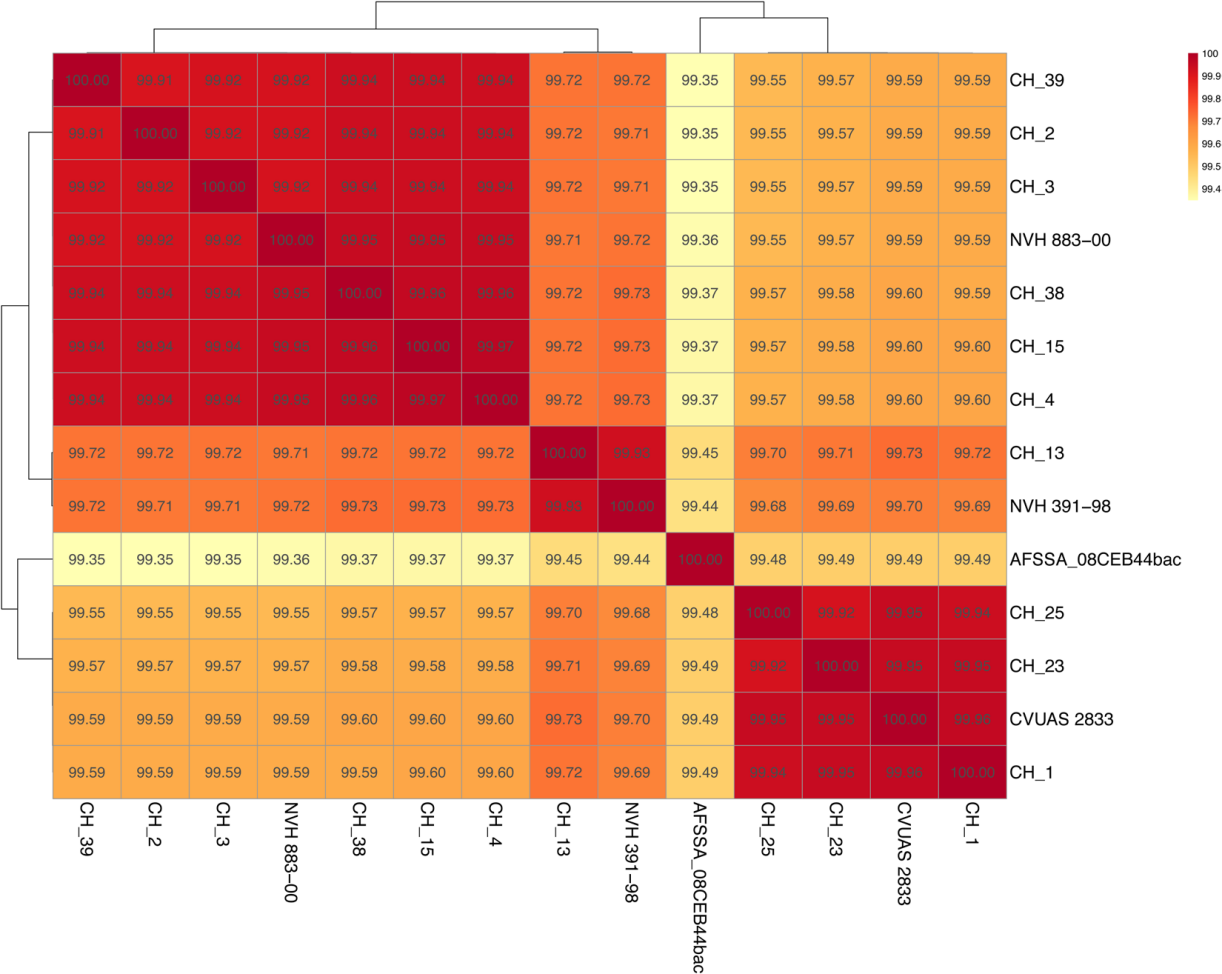
Heat map of the average nucleotide identity (ANI) for strains of the species *B. cytotoxicus*.

An ANI matrix of the 14 strains was constructed and revealed four clades, designated A-D, which differ from each other by at least 0.3% (Fig. 5). Strain AFSSA_08CEB44bac is an outlier, differing at least 0.5% from the other strains.

**Figure 6:**
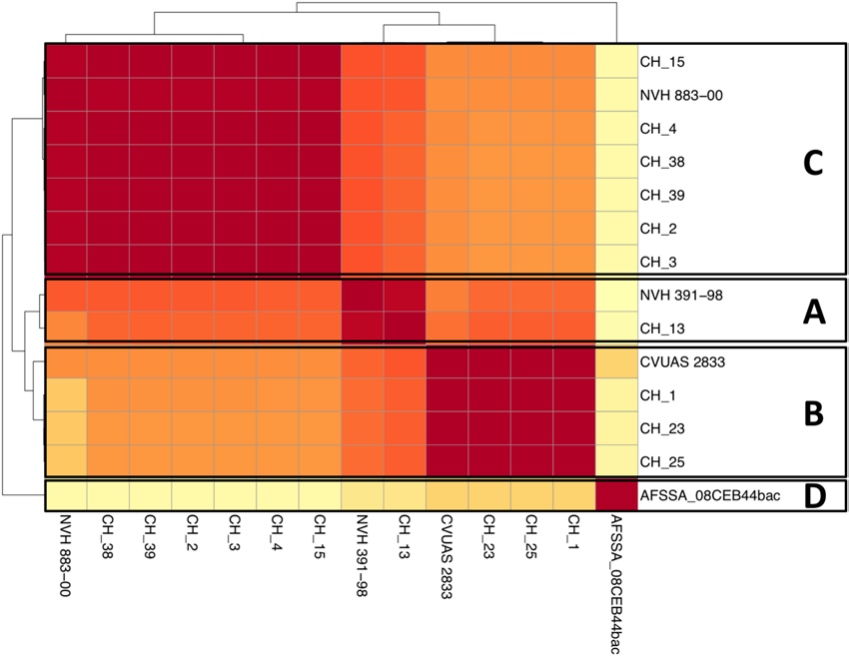
SNP based tree and heat map of the species *B. cytotoxicus*. The four clades are indicated.

In a next step, we compared the number of single nucleotide polymorphisms (SNPs) between the core genomes of the strains (Fig. 6). The strains of clade A (CH_13 and type strain NVH391-98^T^) had 946 SNPs. The strains of clade B (CH_1, CH_23, CH_25, CVUAS 2833) had only ten to 54 SNPs. The strains of clade C either had 12–115 SNPs (CH_2, CH_3, CH_4, CH_38 and CH_39) or 219–227 SNPs (NVH 883–00). Strains in clade A had 7,562 to 9,480 SNPs compared to strains from clade B and 7,426 to 9,806 SNPs compared to strains from clade C (see also Supplementary Table S1). Clade B and C had approximately 10,000 SNPs. Strain AFSSA_08CEB44bac representing clade D had 16,000-20,000 SNPs compared to all other strains. *B. cytotoxicus* has a genome size of approximately 4.1 Mbp and the SNP rates between the clades are therefore 1.8 to 4.8 SNPs per kbp.

**Figure 7:**
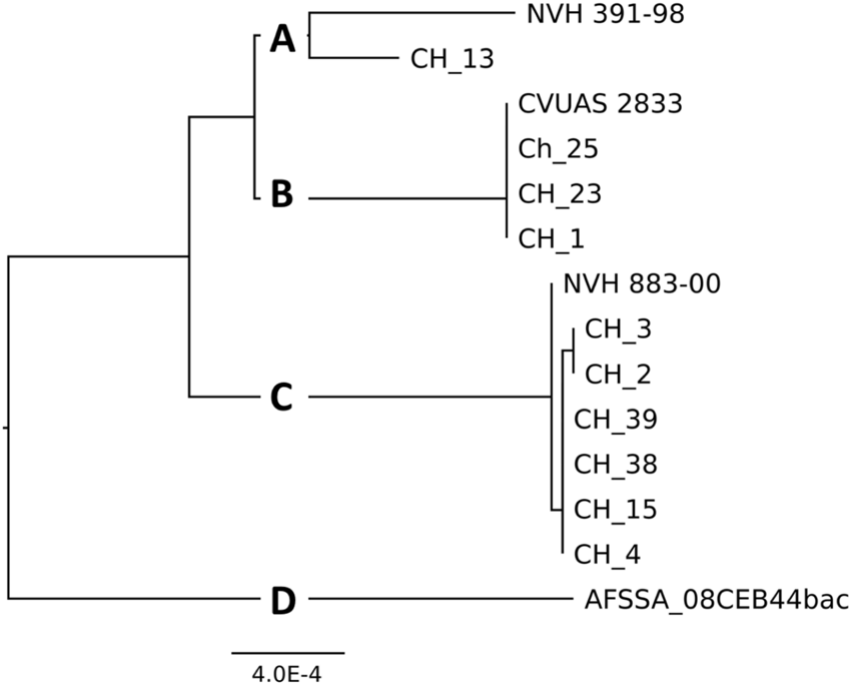
Core genome tree of the species *B. cytotoxicus*.

**Figure 8:**
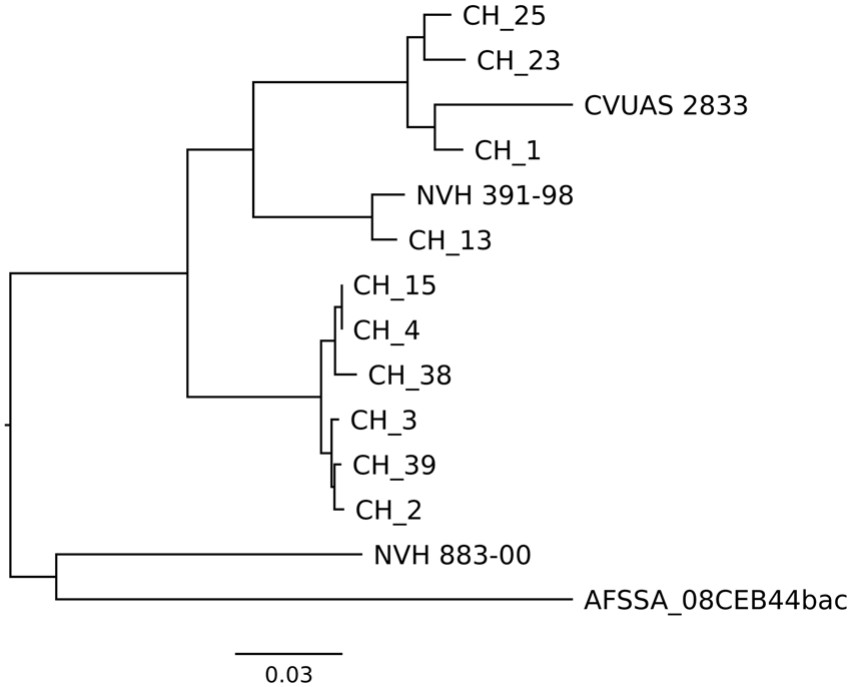
Pan genome tree of the species *B. cytotoxicus*.

A core genome-based tree further confirmed that *B. cytotoxicus* can be differentiated into four clades (Fig. 7). In addition, a pan genome tree is useful to distinguish between strains that are hardly distinguishable in core genome analysis^34^. The pan genome tree of *B. cytotoxicus* strains confirmed the grouping of strains into four clades. However, AFSSA_08CEB44bac and NVH883-00 are now both outliers (Fig. 8).

Taken together, MLST, ANI, and SNP and core-genome analyses, resulted in the same grouping of the 14 *B. cytotoxicus* strains into four clades. Clade A comprised the type strain NVH391-98 and CH_13. Closely related to clade A is clade B, which comprised CH_1, CH_23, CH_25, and CVUAS 2833. Clade C consisted of CH_2, CH_3, CH_4, CH_38, CH_39, and NVH 883-00 and clade D exclusively consisted of AFSSA_08CEB44bac.

### Genetic contents of *B. cytotoxicus*

**Figure 9:**
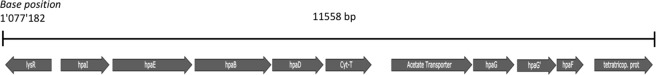
Genetic organization and functional comparison of the *hpa* operon in *B. cytotoxicus* strains. Putative functions are as follows: *lysR* – regulator of the operon; *hpaI* - 2,4-dihydroxyhept-2-ene-1,7-dioic acid aldolase; *hpaE* - 5-carboxymethyl-2-hydroxymuconate semialdehyde dehydrogenase; *hpaD* - 3,4-dihydroxyphenylacetate 2,3-dioxygenase; Cyt-T – cytidyltransferase; acetate transporter - cation acetate symporter; *hpaG* - 2-hydroxyhepta-2,4-diene-1,7-dioate isomerase; *hpaF* - 5-carboxymethyl-2-hydroxymuconate Delta-isomerase; tetratricop. Prot - tetratricopeptide repeat protein. The 330-bp hypothetical gene between Cyt-T and the acetate transporter is not shown.

The core genome of the 14 *B. cytotoxicus* strains contained 3,151 protein encoding genes. The formula of the fitting curve for the core genome dynamics (Supplemental Fig. 1A) predict a decrease of 45 genes after the addition of a 15^th^ genome and therefore the core-genome is still open. Of the 3,151 core genes, 129 were not found in any of the 23 *B. cereus* group genomes used in this study and thus comprise potential *B. cytotoxicus*-specific genes. A functional analysis of *B. cytotoxicus*-specific genes revealed, among others, genes encoding 77 hypothetical proteins, ten transcriptional regulators, 2 CRIPSR cas associated genes and 2 transposases (Table 2). Interestingly, a putative hydroxyphenylalanine (*hpa*) operon was exclusively present in all 14 *B. cytotoxicus* strains. This operon encodes enzymes to degrade the aromatic compound 4-hydroxyphenylacetate to components of the citrate cycle^35,36^. The operon consists of ten genes and a LysR regulator gene is situated directly upstream in the opposite direction (Fig. 9). This parallels the organization in *Escherichia coli*, where the *hpa* regulator is located upstream of the operon^37^. Further, the regulation of operons by an upstream regulator is frequent in bacteria^38^ and LysR is therefore a likely candidate to regulate the *hpa*-operon in *B. cytotoxicus*. A cytidyltransferase is located in the operon, which is not present in the *E. coli* operon. The operon has a GC-content of 39.6%, which exceeds the average GC-content of 35.8% for *B. cytotoxicus* genomes. A search among all *B. cereus* group genomes revealed that nine of the 11 genes of the operon were present in *Bacillus pseudomycoides* strain AFS092012, but not in *B. pseudomycoides* DSM12442, FSL H8-0534 or any of the other 99 *B. pseudomycoides* genomes in the public database. Furthermore, the operon was not detected in genomes of other members of the *B. cereus* group.

**Table 2:** *B. cytotoxicus* core genome genes not found in other genomes of the *B. cereus* group.

Locus in CH_13^a^	Gene in CH_13	Function^b^
CG479_RS05485	CG479_RS05485	2,4-dihydroxyhept-2-ene-1,7-dioic acid aldolase
CG479_RS05500	hpaD	3,4-dihydroxyphenylacetate 2,3-dioxygenase
CG479_RS05515	CG479_RS05515	cytidyltransferase
CG479_RS05535	CG479_RS05535	4-hydroxyphenylacetate isomerase
CG479_RS05530	CG479_RS05530	2-hydroxyhepta-2,4-diene-1,7-dioate isomerase
CG479_RS05525	CG479_RS05525	5-carboxymethyl-2-hydroxymuconate isomerase
CG479_RS15270	CG479_RS15270	glycerophosphodiester phosphodiesterase
G479_RS06185	G479_RS06185	LTA synthase family protein
CG479_RS07295	CG479_RS07295	transcriptional regulator
CG479_RS07395	CG479_RS07395	sporulation protein YjcZ
CG479_RS07860	CG479_RS07860	TetR/AcrR family transcriptional regulator
CG479_RS07990	CG479_RS07990	thiamine biosynthesis protein ThiF
CG479_RS08090	CG479_RS08090	GbsR/MarR family transcriptional regulator
CG479_RS08120	CG479_RS08120	aspartate aminotransferase family protein
CG479_RS09375	CG479_RS09375	spore germination protein
CG479_RS09435	CG479_RS09435	methyl-accepting chemotaxis protein
CG479_RS10095	CG479_RS10095	transcriptional regulator
CG479_RS10100	CG479_RS10100	ImmA/IrrE family metallo-endopeptidase
CG479_RS10240	CG479_RS10240	DMT family transporter
CG479_RS10490	CG479_RS10490	MarR family transcriptional regulator
CG479_RS10975	cas2	CRISPR-associated endonuclease Cas2
CG479_RS10980	cas4	CRISPR-associated protein Cas4
G479_RS11880	G479_RS11880	acyltransferase
CG479_RS11890	CG479_RS11890	N-acetyltransferase
CG479_012025	CG479_012025	phosphonate ABC transporter permease
CG479_RS12175	CG479_RS12175	transcriptional regulator
CG479_RS12350	CG479_RS12350	IS30 family transposase
G479_RS12365	G479_RS12365	MATE family efflux transporter
CG479_RS12655	CG479_RS12655	IS256 family transposase
CG479_RS12690	CG479_RS12690	ArsR family transcriptional regulator
CG479_RS12835	CG479_RS12835	enterotoxin
G479_RS15125	G479_RS15125	transcriptional regulator
CG479_RS15995	CG479_RS15995	MFS transporter
CG479_RS17000	CG479_RS17000	non-ribosomal peptide synthetase
G479_RS18300	G479_RS18300	S-layer protein
CG479_RS18315	CG479_RS18315	ArsR family transcriptional regulator
CG479_RS18510	CG479_RS18510	YjcZ family sporulation protein
CG479_RS18930	CG479_RS18930	NupC/NupG family nucleoside CNT transporter
CG479_RS20300	CG479_RS20300	NADH-quinone oxidoreductase subunit C
CG479_RS20520	CG479_RS20520	DNA-directed RNA polymerase subunit delta
CG479_RS21035	CG479_RS21035	transcriptional antiterminator BglG
CG479_RS01030	CG479_RS01030	CPBP family intramembrane metalloprotease
CG479_RS01060	CG479_RS01060	MFS transporter
CG479_RS02825	CG479_RS02825	histidine kinase
CG479_RS02835	CG479_RS02835	amidohydrolase family protein
CG479_RS02890	CG479_RS02890	alpha/beta hydrolase
CG479_RS03705	CG479_RS03705	histidine kinase
CG479_RS04625	CG479_RS04625	LytR family transcriptional regulator
CG479_RS05280	CG479_RS05280	spore coat protein

^a^strain CH_13 was used as reference strain, ^b^hypothetical genes were omitted.

In addition, the pan genome of the 14 *B. cytotoxicus* strains consisted of 5,111 genes. Dynamics of the pan genome resulted in a fitting curve with an α ≈ 0.11, indicating an open pan genome. Remarkably, the three draft genomes in the dataset, i.e. AFSSA_08CEB44bac, NVH 883-00, and CVUAS 2833, contained a high number of unique genes: 335, 204, and 64 respectively (Table 3). Moreover, AFSSA_08CEB44bac and NVH 883-00 uniquely shared 40 genes, mainly encoding phage-related or hypothetical functions. All other strains exhibited less than seven unique genes. Remarkably, if the 40 genes shared solely by AFSSA_08CEB44bac and NVH 883-00 are deleted from the pan-genome matrix, the strains do not cluster together anymore in a pan genome tree (data not shown).

**Table 3:** Comparison of the characteristics of all *B. cytotoxicus* strains, which were either full genomes sequenced in the course of this study (marked by an asterisk) or complete genome sequences that were already publicly available.

Strain	Source	Genes					Plasmids
		Total	Accessory	Unique	Exclusively absent	Virulence factors	
CH_1*	Mashed potatoes, Switzerland, 2014	3866	691	2	7	264	14 kb, 53 kb, 67 kb
CH_2*	Mashed potatoes, Switzerland, 2014	3981	794	0	9	262	53 kb, 83 kb
CH_3*	Mashed potatoes, Switzerland, 2014	3984	796	1	1	263	53 kb, 83 kb
CH_4*	Mashed potatoes, Switzerland, 2014	3943	766	0	0	260	83 kb
CH_13*	Mashed potatoes, Switzerland, 2014	3837	664	2	13	264	79 kb
CH_15*	Mashed potatoes, Switzerland, 2014	3943	766	0	0	260	83 kb
CH_23*	Mashed potatoes, Switzerland, 2014	3888	696	5	37	259	53 kb, 67 kb
CH_25*	Mashed potatoes, Switzerland, 2014	3926	712	4	23	259	53 kb, 67 kb
CH_38*	Mashed potatoes, Switzerland, 2014	3922	742	0	12	262	53 kb, 83 kb
CH_39*	Mashed potatoes, Switzerland, 2014	3989	799	0	5	264	83 kb
AFSSA_08CEB44bac	Cooked semolina, France, 2008,	4218	840	335	35	268	—
CVUAS-2833	Mashed potatoes linked to foodborne illness, Germany, 2007	3782	579	64	93	266	—
NVH391-98^T^	Vegetable puree, France, 1998	3844	663	6	6	265	7 kb
NVH 883-00	Spices, Norway, 2000	4254	1014	222	12	259	—

The low abundance of unique genes in most strains might be caused by high similarity of the strains; thus, we carried out an in-depth analysis of the unique genes detected. Strains NVH 883-00 and CVUAS 2833 were omitted because of their high number of unique genes. Strains NVH 391-98, CH_13, CH_1, CH_23, and CH_25 of clades A and B had 73 genes not found in other clades. Functional analyses revealed 39 hypothetical proteins, whereas other functions were mainly related to phages (see Supplementary Table S2). The strains of clade C (CH_2, CH_3, CH_4, CH_15, CH_38, and CH_39) had 167 clade-specific genes, with 115 representing hypothetical proteins. In addition, a lactoylglutathione lyase and a glutathione transferase were found. Both enzymes might be involved in methylglyoxal detoxification. Further, an operon encoding the functions to convert mannitol to fructose with parallel conversion of mannonate to pyruvate was present.

### Genes encoding virulence factors and toxins in *B. cytotoxicus*

In a next step, the virulence of the *B. cytotoxicus* strains was assessed via a comparison to the virulence factor database VFDB^39^. The strains had between 259 and 268 putative virulence factors (Table 2), with 220 factors being found in all 14 strains. Strains NVH 883-00, CH_23, and CH_25 had the fewest virulence factors (n = 259). Strain NVH 883-00 was missing genes present in all other *B. cytotoxicus* strains that encode three putative virulence factors: a collagen adhesion protein, the motility protein MotA, and a gene involved in non-ribosomal peptide synthesis. Strain AFSSA_08CEB44bac harbored six genes not found in any other strain, including a gene coding for subtilisin.

The *cytK-1* gene characteristic for *B. cytotoxicus* exhibited one amino acid substitution, T257A, between strains from clade A, B, D and clade C. This change is next to the conserved residue Y256^24^.

### Plasmids of *B. cytotoxicus*

**Figure 10:**
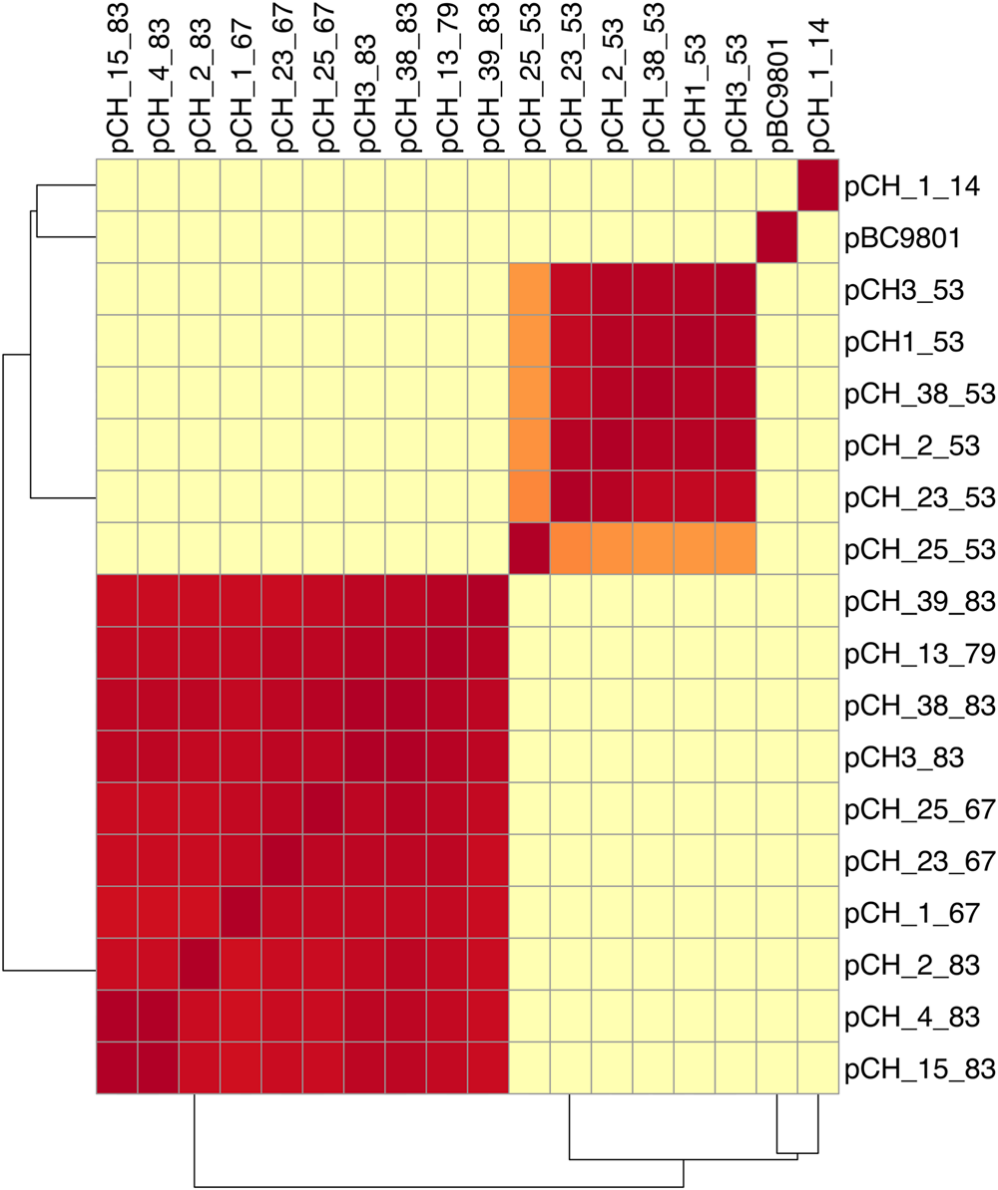
ANI analyses of plasmids in *B. cytotoxicus*. The plasmids are indicated with the respective strain identifier followed by the size in kb.

The 11 completely assembled genomes of *B. cytotoxicus* each contain one or more plasmids. No plasmid-like sequences were identified in the draft genomes of strains AFSSA_08CEB44bac, NVH 883-00, and CVUAS 2833. In total, 18 plasmids with sizes from 7 to 83 kb were identified (Table 2). The plasmids can be divided into four groups that exhibit no sequence similarity (Fig. 10). The first group contains ten plasmids of 83 kb, 79 kb, and 67 kb, the second group contains six plasmids of 53 kb, and the final two groups each contain one plasmid of 14 kb and 7 kb, respectively. The first group of plasmids was found throughout the ten strains sequenced in this study, i.e. CH_1 to CH_39 (Table 2). Remarkably, the plasmid size was 79 kb in clade A, 67 kb in clade B and 83 kb in clade C. Additional genes on the 83 kb plasmids encoded one conjugational protein and 12 hypothetical proteins. The 83 kb plasmid group had an average nucleotide identity of >99.8% with a coverage of virtually 100%. The maximum nucleotide identity with other *Bacillus* plasmids was 79%, but with a maximum coverage of 56% as revealed by a blastN search against the NCBI nucleotide database. The 53 kb plasmids had an average nucleotide identity of >99.7% with a coverage of virtually 100%. The plasmids were similar to the plasmid pBCM1301 from *Bacillus cereus* M13 with 97% identity and a coverage of 86%.

The 18 plasmids encoded 1,258 identified genes encoding 52 different functions. The complete list can be found online as Supplementary Table S3. The 67–83 kb plasmids carried genes coding for proteins with conjugational functions, stress proteins, transposons, a peptide transporter and a sulfite exporter. A toxin-antitoxin system of the type III ToxN/AbiQ family toxin-antitoxin system was also present on these plasmids. Homologs of this system with up to 94.2% amino acid identity were found in several plasmids of the *B. cereus* group. The 53 kb plasmid-encoded genes associated with stress-related proteins and transposons. Genes conferring resistance to antibiotic agents or encoding virulence factors were not identified on any of the plasmids.

## Discussion

*B. cytotoxicus* is a recently described species that causes severe foodborne illness^1^. This study presents the phylogeny and genome content of *B. cytotoxicus* and provides novel clues on the evolution of the species. Complete genome-based phylogeny revealed that *B. cytotoxicus* is a distinct species within the *B. cereus* group, consistent with results obtained for the genome of type strain NVH 391-98^17^. The ANI and core genome-based trees of the *B. cereus* group indicate that *B. cytotoxicus* is – like *B. anthracis* – a relatively homogenous species, which is in stark contrast to other members of the *B. cereus* group such as *B. cereus* and *B. thuringiensis*, which exhibit a rather heterogeneous population structure^40,41^. While the pan genome-based tree showed a higher degree of evolutionary divergence (Fig. 1), this is in agreement with the generally higher divergence exhibited by pan genome-based trees compared to core genome-based trees^34,42^. The core genome-based tree containing only *B. cytotoxicus* strains also depicted the species as less homogenous, possibly because it was based on a higher number of genes than the *B. cereus* group core genome tree.

The heterogeneity was reflected in clustering in four clades. The clades were already visible in MLST analysis but based on only one or two SNPs. The genome-based trees in this study are far more reliable, as the number of SNPs between the clades is at least 7,428. Further, the SNP rate is 300-fold higher than the sequencing error rate for Pacbio SMRT sequencing (https://www.pacb.com/uncategorized/a-closer-look-at-accuracy-in-pacbio/) and complete genome trees therefore represent a valid hypothesis for evolution of *B. cytotoxicus*. In addition, as leading evolutionary biologist Ernst Mayr pointed out, evolution occurs in a complete organism and not on a single gene (https://www.edge.org/conversation/ernst_mayr-what-evolution-is) and hence complete genomes are preferred for phylogenetic analyses.

The SNP based analysis has the highest resolving power as it is based on DNA sequences and takes into consideration intergenic regions. The core-genome-based analysis is based on protein sequences and ignores intergenic regions and silent mutations. Nevertheless, the four clades also appeared in the core-genomes tree, showing that their distinction is robust.

The occurrence of four clades in core, pan, and SNP-based trees (Fig. 2) indicates that the clades are results of true evolutionary events. Strain NVH_883-00 can be found in clade C in the MLST, SNP and core genome trees, but clusters close to AFSSA_08CEB44bac in the pan genome tree (Fig. 2). Such differences can be considered as alternative evolutionary hypotheses^42^. NVH 883-00 shared many phage genes with AFSSA_08CEB44bac and their close clustering in the pan genome tree, which was based on a presence-absence matrix, was due to the high amount of uniquely shared genes. In addition, the presence of these genes in the two strains suggests that horizontal gene transfer mediated via phages occurred between the two strains.

The species *B. cytotoxicus* harbors a complete hydroxyphenylalanine operon that is absent in all other *B. cereus* group members and encodes the machinery to degrade aromatic compounds to succinate and pyruvate^35^. The pathway is normally associated with soil bacteria and is also known to contribute to *para*-cresol production out of tyrosine in the human large intestine^43,44^. Lignin-derived aromatic compounds are a major carbon source in soil and the capability to degrade these compounds is present in many soil bacteria^36^. In addition, free amino acids are released in the human gut due to the activity of peptidases and proteases from human and bacterial origin^45^. The presence of the *hpa*-operon suggests that *B. cytotoxicus* can utilize aromatic compounds, including tyrosine as carbon source. Furthermore, Gram-negative bacteria are susceptible to *para*-cresol and production of *para*-cresol in the intestine leads to reduced numbers of Gram-negatives^46^. The *hp*a-operon thus provides a clear evolutionary advantage for *B. cytotoxicus* through access to an additional carbon source and potentially also through production of the inhibiting agent *para*-cresol. However, the activity of the *hpa*-operon and its role in the metabolism of *B. cytotoxicus* remains to be elucidated. The presence of the operon points towards soil as the ecological niche of *B. cytotoxicus*, which is consistent with isolation of the organism almost exclusively from potato products^23,32^. Soil may represent the source of contamination for mashed potatoes after detecting *B. cytotoxicus* on a raw potato^32^.

The substantially higher GC content of the operon compared to the rest of the chromosome suggests that it was acquired from a higher GC% organism, likely another soil bacterium. The operon might have given a *B. cytotoxicus* ancestor an evolutionary advantage through utilization of tyrosine. This evolutionary advantage eventually resulted in species differentiation. The presence of a cytidyltransferase even suggests that energy can be gained in this pathway via CTP dependent substrate-level phosphorylation.

The clade-specific amino acid substitution in CytK-1 next to a conserved residue might lead to differences in the activity of CytK-1 and hence virulence. Type strain NVH 391-98^T^ that caused fatal cases of foodborne disease in an outbreak in France and CVUAS 2833 linked to foodborne disease in Germany clustered in clade A and B, respectively. This supports the hypothesis that strains from these clades could be more virulent. Strain NVH 883-00 is non-cytotoxic^26^ and belongs to clade C. This is consistent with the hypothesis that strains from clade C exhibit low toxicity. Furthermore, virulence factor analyses revealed that NVH 883-00 uniquely lacks three virulence factors, the flagellar motor MotA, a collagen adhesion protein, and a gene involved in non-ribosomal peptide synthesis. The low virulence of this strain may therefore also be attributed to the deficiency in these three factors. Cytotoxicity and virulence studies are needed to provide comprehensive data supporting or dismissing a link between the virulence potential and the different clades.

Clade C possesses unique genes encoding two functions, a lactoylglutathione system and a mannitol conversion system. Both may give clade C strains an advantage; the first to survive methylglyoxal, the second to regenerate NAD^+^. The latter reaction is useful if redox regeneration is limited, and is used by fermentative bacteria^47,48^.

The distribution of plasmids in strains from the different clades suggests horizontal gene transfer in *B. cytotoxicus*, at least between strains from the same geographical region (Switzerland). The 53 kb plasmids were solely found in clade C. Further, the 53 kb plasmids had higher similarity to plasmids of the *B. cereus* group than to the 83 kb plasmids. This higher similarity strongly suggests that the 53 kb plasmids were acquired by clade C strains after the 83 kb plasmids were acquired and after differentiation into clades occurred. The encoded stress proteins may handle stress linked to conjugation, rather than providing the host with increased tolerance when faced with environmental stress^49^. The toxin-antitoxin system is one commonly found on *B. cereus* plasmids and ensures plasmid maintenance during germination and sporulation^37^.

## Conclusion

We were able to show that, in contrast to some other *B. cereus* group species, *B. cytotoxicus* strains are phylogenetically distinct. Genomic content analysis revealed that a hydroxyphenylalanine (*hpa*) operon and its putative regulator are present in *B. cytotoxicus*, but absent in all other members of the *B. cereus* group. This operon codes for the machinery to degrade aromatic compounds to succinate and pyruvate and was likely acquired from another *Bacillus* species. It allows for utilization of tyrosine and might have given a *B. cytotoxicus* ancestor an evolutionary advantage that eventually resulted in species differentiation. Plasmid content of the strains showed that *B. cytotoxicus* is flexible in exchanging genes, allowing for quick adaptation to the environment. Genome-based phylogenetic analysis divided the investigated *B. cytotoxicus* strains into four clades that also differed in virulence gene content. In conclusion, our results provide novel insights essential to extend the currently very limited understanding of *B. cytotoxicus* virulence, ecology, and evolution.

## Methods

### Bacterial strains

Ten *B. cytotoxicus* strains (CH_1, CH_2, CH_3, CH_4, CH_13, CH_15, CH_23, CH_25, CH_38, CH_39) were isolated from mashed potatoes, which had been collected from regiment kitchens of the Swiss Army in 2014 and 2015. For bacterial isolation, ten-fold dilution series of the samples in 0.85% NaCl were streaked on MYP agar (Oxoid) and incubated at 37 °C over night. Plates were subsequently checked for colonies exhibiting a mannitol-negative and egg-yolk lecithinase-positive phenotype and a colony morphology consistent with the strains of the *B. cereus* group.

In addition, complete genome sequences of various reference strains were downloaded from the NCBI database in January-May 2018. A comprehensive list of all strains used in this study is provided as Table 1.

### Complete genome sequencing

Genomic DNA was extracted and purified from *B. cytotoxicus* strains CH_1, CH_2, CH_3, CH_4, CH_13, CH_15, CH_23, CH_25, CH_38, CH_39 using the GenElute Bacterial Genomic DNA Kit (Sigma, Buchs, Switzerland). Genomes were sequenced on a PacBio RS II sequencer (Pacific Biosciences, Menlo Park, USA) using the single-molecule real-time sequencing technology (SMRT) chemistry at the Functional Genomics Centre Zurich. Sequencing each sample on two SMRTcells with P6/C4 chemistry and 180-minute movies generated 102,934 to 170,329 sequence reads with a mean read length of 10,120 to 18,569 bp, corresponding to approximately 200 to 600-fold genome coverage, depending on the sample. PacBio sequences were assembled de novo using the SMRT Analysis v2.3.0 software and the Hierarchical Genome Assembly Process (HGAP_3) workflow. Annotation of the genomes was carried out using the NCBI Prokaryotic Genome Automatic Annotation Pipeline (PGAAP; http://www.ncbi.nlm.nih.gov/genome/annotation_prok/)^50^.

### General genome analyses

Core and pan genomes were identified using the USEARCH algorithm^51^ at a 75% sequence identity cut-off within the Bacterial Pan Genome Analysis (BPGA) software package. The dynamics of the core and pan genome were plotted and a fitting curve calculation was performed using a power fit for the pan genome and an exponential fit for the core genome, both embedded in BPGA (supplemental Fig. 1). If the dynamics of the pan genome result in a fitting curve with an α < 1, the pan genome is considered open^52^.

Average nucleotide identity (ANI) was calculated using the perl script “get_homologous”^42^ and calculated using the following settings: -E < 1e-05 for BLAST searches and -C 75% minimum alignment coverage. The options “-A” to produce a tab-separated file with relative average sequence identity and the option “-a “CDS” “ to run BlastN on the CDS nucleotide sequences were triggered to obtain an ANI matrix file. The ANI matrix was visualized in a heat map using clustvis^53^, with a Manhattan distance calculation and a complete linkage for rows and columns.

The ANI for plasmids was calculated using the python script average_nucleotide_identity.py in the pyani suite available at, https://github.com/widdowquinn/pyani. It calculates the ANI according to Richter^54^. In short, it aligns sequences using nucmer in Mummer^55^ and uses TETRA^56^ to calculate nucleotide frequencies. Nucleotide fasta files were used as input.

### Phylogeny methodology

Core and pan genomes were identified using USEARCH^51^ at a 75% sequence identity cut-off within the Bacterial Pan Genome Analysis tool (BPGA) software package^57^. Maximum likelihood trees of the core genomes were constructed based on MUSCLE^58^ alignments of the concatenated core proteins and performed in BPGA. Pan genome trees were constructed from a presence-absence matrix (1/0) from orthologous clusters using standard settings in BPGA. The resulting Newick files were visualized as midpoint rooted trees in Figtree 1.4.3 (http://tree.bio.ed.ac.uk/software/figtree/).

MLST-based trees were obtained by aligning the concatenated nucleotide sequences using MAFFT^59^. The alignment was converted to a Newick format using the nearest neighbor joining method based on the Jukes-Cantor substitution model and a midpoint-rooted tree was visualized in FigTree 1.4.3. Phylip format tree files are available in Supplementary Material S4.

### Single nucleotide polymorphisms analysis

Single nucleotide polymorphisms (SNPs) were identified using Parsnp and Gingr in the Harvest suite^60^. SNPs were first identified with Parsnp using genomic nucleotide fasta files as input data. All 14 *B. cytotoxicus* genomes were compared to each *B. cytotoxicus* genome as reference genome. Output data files were converted to variant calling files using Gingr in the harvest suite^60^. The number of SNPs was calculated as being the sum of the variants compared to the reference strain. A matrix was produced with the SNPs per strain. The matrix was hierarchically clustered using complete linkage and Manhattan distance in R (R-project.org) using an in-house R-script. A heatmap was produced using Clustvis^53^.

### Genetic content analyses

Pan genome gene presence-absence matrices were produced using get_homologous^42^: First, a cluster of orthologous groups (COG) and an orthologous Markov clustering (OMCL)-based pan genome was determined using the following settings: -E < 1e-05 for blast searches, -C 75% minimum alignment coverage, and -t 0 for obtaining all clusters. Subsequently, a pan genome matrix was produced using the script compare_clusters.pl using the OMCL and COG-based pan genomes as input and the option -m to obtain a matrix^42^. This method is more stringent than the BPGA method, as it builds a matrix based on both OMCL and COG and thus reduces the number of false positives in the gene content analysis. The matrix was converted to a presence-absence matrix (1/0) in Microsoft’s Excel (Microsoft, Redmond, WA, USA) to determine gene content.

### Identification of genes encoding putative virulence factors

Virulence factors were identified by comparing the predicted proteome of each strains to the large protein set B of the virulence factor database VFDB, which contains 26,594 protein sequences, including 397 from the *B. cereus* group^39^, downloaded April 3^rd^, 2018. A bidirectional best-hit approach using blastp^61^ was applied to minimize the number of false positive hits. Blast settings were E < 1e-05 and an additional cut-off of 75% minimum alignment was used.

### Accession numbers

Sequence and annotation data of the complete genomes of *B. cytotoxicus* strains CH_1, CH_2, CH_3, CH_4, CH_13, CH_15, CH_23, CH_25, CH_38, and CH_39 are deposited in the GenBank database under the accession numbers listed in Table 1.

## Electronic supplementary material

Supplementary Dataset 1

Supplementary Dataset 2

Supplementary Dataset 3

Supplementary Dataset 4

## Data Availability

The datasets supporting the conclusions of this article are available in the GenBank repository. For accession numbers see Table 1.
