# Supplementary material for: Whole-genome-based phylogeny of Bacillus cytotoxicus reveals different clades within the species and provides clues on ecology and evolution
Source: Sci Rep. 2019 Feb 13;9:1984. doi: 10.1038/s41598-018-36254-x (PMC6374410; doi:10.1038/s41598-018-36254-x)

Whole-genome-based phylogeny of *Bacillus cytotoxicus* reveals different clades within the species and provides clues on ecology and evolution

Marc J. A. Stevens[1], Taurai Tasara[1], Jochen Klumpp[2], Roger Stephan[1], Monika Ehling-Schulz[3], Sophia Johler[1,*]

Supplemental Figure 1: A) Dynamics of the core genome of 14 *B. cytotoxicus* strains and the formula resulting from curve fitting. B) Dynamics of the pan genome of 14 *B. cytotoxicus* strains.

A)

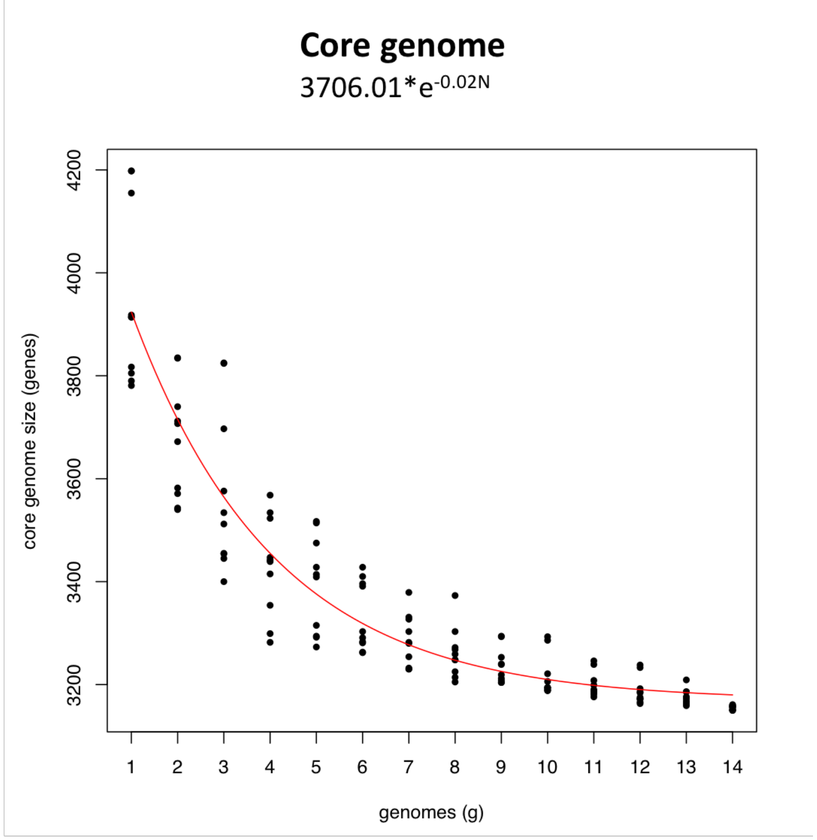

B)

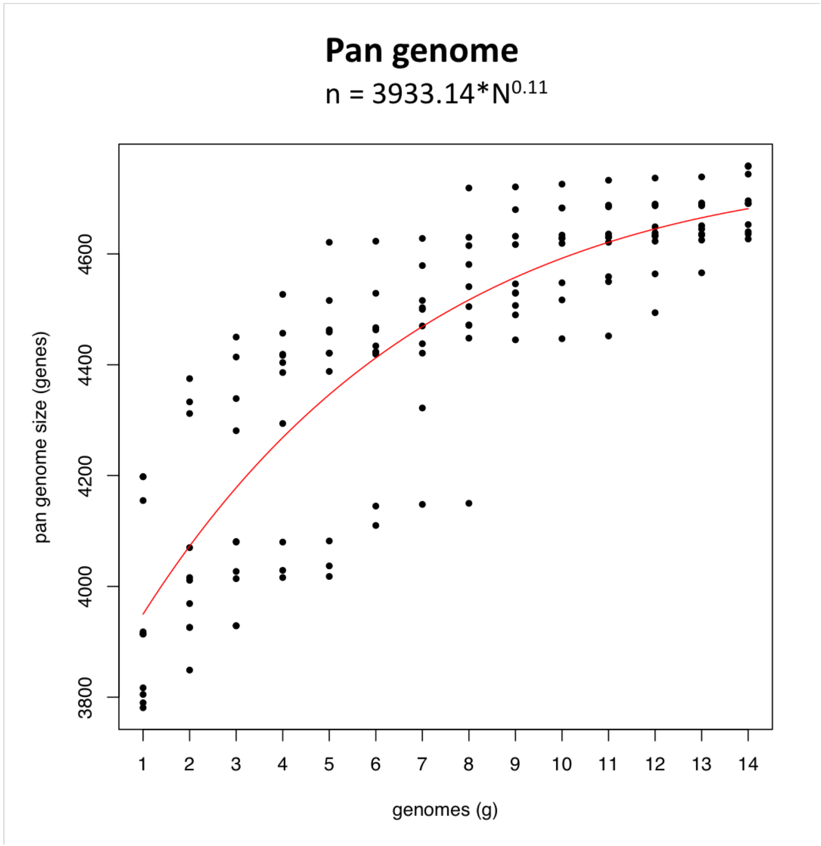

Supplement: Supplementary file 1 — Supplementary Dataset 1 [file 41598_2018_36254_MOESM1_ESM.pdf]
